# Activation and Disproportionation of Zr_2_Fe Alloy as Hydrogen Storage Material

**DOI:** 10.3390/molecules24081542

**Published:** 2019-04-19

**Authors:** Jiangfeng Song, Jingchuan Wang, Xiaoyu Hu, Daqiao Meng, Shumao Wang

**Affiliations:** 1 Institute of Atomic and Molecular Physics, Sichuan University, Chengdu 610065, China; iterchina@163.com; 2 Institute of Materials, Chinese Academy of Engineering Physics, Jiangyou 621908, China; wangjingchuan@caep.cn (J.W.); mengdaqiao@caep.cn (D.M.); 3 School of Resource & Environment and Safe Engineering, University of South China, Hengyang 421001, China; 13990105175@163.com; 4 Energy Materials and Technology Research Institute, General Research Institute for Nonferrous Metals, Beijing 100088, China

**Keywords:** Zr_2_Fe, activation, disproportionation, hydrogen isotope

## Abstract

As a hydrogen storage material, Zr_2_Fe alloy has many advantages such as fast hydrogen absorption speed, high tritium recovery efficiency, strong anti-pulverization ability, and difficulty self-igniting in air. Zr_2_Fe alloy has lower hydrogen absorption pressure at room temperature than LaNi_5_ alloy. Compared with the ZrVFe alloy, the hydrogen release temperature of Zr_2_Fe is lower so that the material can recover hydrogen isotopes at lower hydrogen concentration efficiently. Unfortunately, the main problem of Zr_2_Fe alloy in application is that a disproportionation reaction is easy to occur after hydrogen absorption at high temperature. At present, there is little research on the generation and influencing factors of a disproportionation reaction in Zr_2_Fe alloy. In this paper, the effects of temperature and hydrogen pressure on the disproportionation of Zr_2_Fe alloy were studied systematically. The specific activation conditions and experimental parameters for reducing alloy disproportionation are given, which provide a reference for the specific application of Zr_2_Fe alloy.

## 1. Introduction

A tritium safety containment system plays an important role in the fusion reactors. The recovery of tritium from tailed helium gas is an indispensable technology for the efficient use of tritium. For the Zr-based alloy-filled flow bed in the inert gas environment, the hydrogen and its isotope absorption efficiency is extremely high, which fulfils the performance requirements as recovery materials [1,2,3,4,5]. In particular, Zr_2_Fe alloy has the advantages of fast hydrogen absorption rate and high absorption efficiency. Compared with those of the high concentration hydrogenation materials [6,7,8,9], the equilibrium pressure at room temperature is around 10^−6^ Pa for Zr_2_Fe alloy [10]. Zr_2_Fe alloy has high feasibility and safety for the application of hydrogen storage and treatment. However, the Zr-based alloy is easy to disproportionate during high-temperature operation, which greatly reduces the storage capacity of the alloy, and is not conducive to repeated cycle applications. Hara et al. [11] carried out a lot of research on the hydrogen disproportionation reaction of Zr_2_M series alloys, and found that Zr_2_M series alloys can form stable Zr_2_MH_5_ (M = Ni, Co, Fe) hydrides. Otherwise, the alloy is prone to disproportionation. Especially when the temperature is raised to 773 K, the disproportionation reaction of this alloy proceeds very quickly, and the ZrH_2_ phase is formed within minutes. At this temperature, ZrCo, ZrNi, and other alloys also disproportionate, however, the reaction rate is significantly slower. Zr_2_Fe alloy is more prone to disproportionation than other alloys. Prigent et al. [12] measured the cyclic properties of Zr_2_Fe alloy at 0.10 MPa under pure hydrogen conditions. The results showed that the hydrogen absorption capacity was significantly affected by the number of cycles. When hydrogen is absorbed for the first time, the hydrogen absorption capacity is about 1.88 wt %. After hydrogen evolution at 350 °C for 3 h, the second hydrogen absorption capacity becomes 1.18 wt %. Under the same conditions, the hydrogen capacity is only 0.84 wt % after third cycle. The decreased hydrogen storage capacity can directly reflect the extent of the disproportionation reaction. Roupcová et al. [13] also found that the zirconium-rich phase Zr_2_Fe in the structure is more prone to disproportionation after hydrogen absorption. Temperature and pressure are the most commonly used system control parameters in applications, but are rarely reported in literature. In this paper, the activation method of Zr_2_Fe alloy and the disproportionation effect of different temperature and hydrogen partial pressure on Zr_2_Fe alloy were studied systematically. The specific activation parameters and the method to reduce the disproportionation of the alloy are given below.

## 2. Results and Discussions

### 2.1. Optimization of Activation Parameters of Zr_2_Fe Alloy

**Figure 1:**
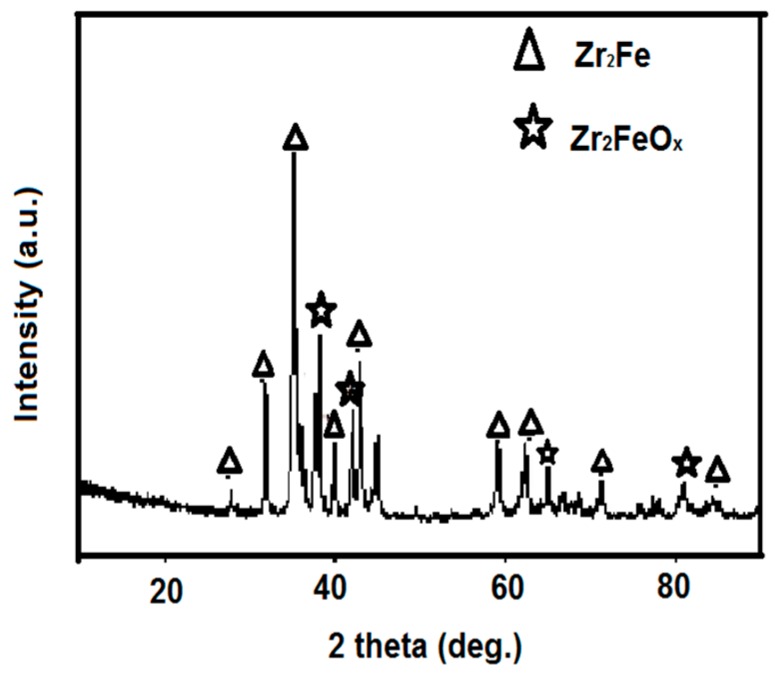
X-ray power diffraction (XRD) pattern of Zr_2_Fe alloy.

XRD patterns of the as-received Zr_2_Fe alloy without any handling are shown in Figure 1. Zr_2_Fe was successfully obtained and presented a polycrystalline structure. Unfortunately, the diffraction peak of Zr_2_FeO_x_ in powder was observed in the XRD pattern, which was the reason for activation prior to use.

#### 2.1.1. Optimization of Activation Temperature

**Figure 2:**
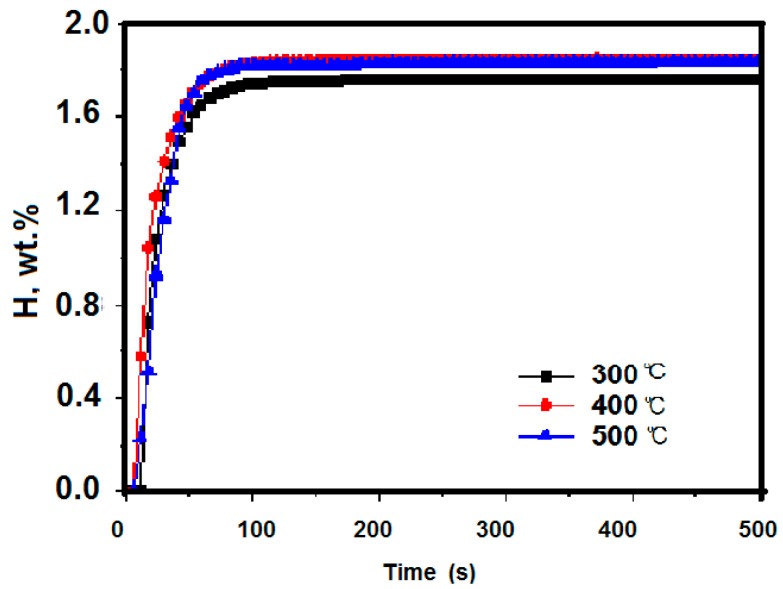
Kinetics of hydrogen absorption at 0.1 MPa after 3 h activation at different temperatures.

The as-received Zr_2_Fe samples were firstly activated and made to form Zr_2_Fe hydride under H_2_ with 0.1 MPa at different temperatures (300 °C, 400 °C, and 500 °C) for 3 h. Figure 2 shows the hydrogen absorption kinetic curves for samples after activation. It can be seen that the samples at different activated conditions achieve hydrogen saturation essentially within 100 s and the most hydrogen absorption of tested samples are 1.765 wt %, 1.848 wt %, and 1.836 wt % at 300 °C, 400 °C, and 500 °C, respectively. Some interesting phenomena were discovered. It was clear that the amount of hydrogen absorption for samples handled at 400 °C and 500 °C was astonishingly greater than when activated at 300 °C, indicating that activation temperature above 400 °C was required to reach complete activation for Zr_2_Fe alloy. Meanwhile, maximum hydrogen absorption and kinetic trends of samples activated under 400 °C and 500 °C is similar in Figure 2, indicating that the effects of raising temperature to 500 °C on an activated sample are weak and the Zr_2_Fe alloy could be completely activated at 400 °C in a vacuum.

**Figure 3:**
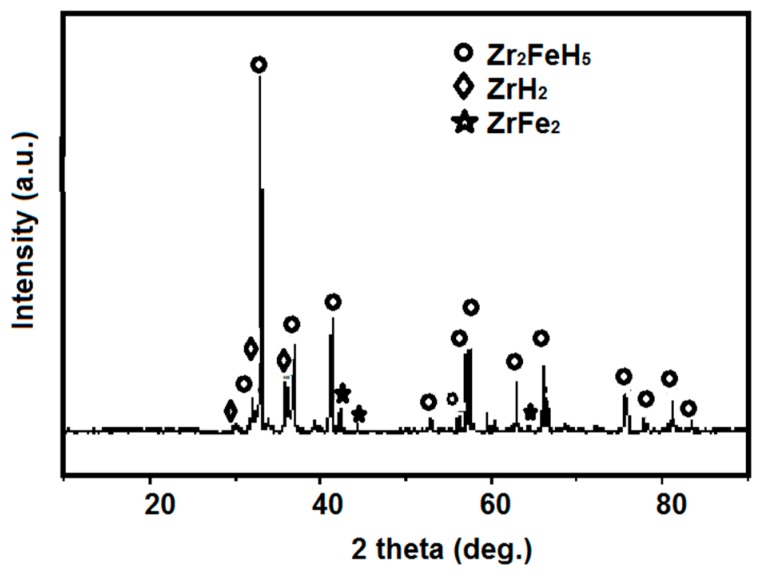
XRD patterns of the hydrogenated product of Zr_2_Fe alloy.

The crystal structure of activated Zr_2_Fe alloy after hydrogenation at room temperature was carried out by X-ray diffraction, as shown in Figure 3. It is clear that Zr_2_FeH_5_ is considered a principle phase for Zr_2_Fe hydride at room temperature, and a small impurity phase is also observed, such as ZrH_2_ and ZrFe_2_, indicating that a part of the Zr_2_FeH_5_ phase is disproportionated firstly at hydrogen absorption. The hydrogen absorption and disproportionation reaction of Zr_2_Fe is shown below:

2Zr_2_Fe+5H_2_↑→2Zr_2_FeH_5_

2Zr_2_FeH_5_→3ZrH_2_+ZrFe_2_+2H_2_↑

#### 2.1.2. Optimization of Activation Treatment Time

**Figure 4:**
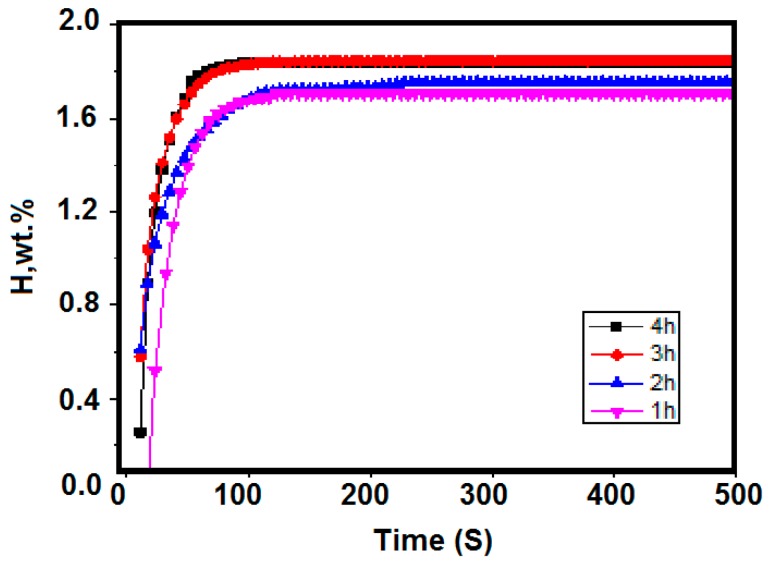
Hydrogen absorption kinetics curves at different activation times of 400 °C at 0.1 MPa.

**Figure 5:**
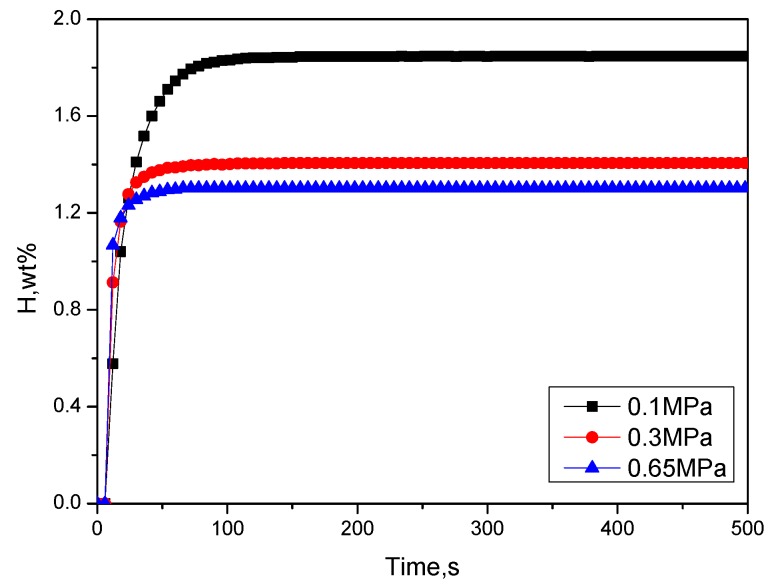
Hydrogen absorption kinetics curves at room temperature and different pressures after activating Zr_2_Fe at 400 °C for 3 h.

The activation temperature was taken at 400 °C and the activation time took 1 to 4 h. The corresponding room temperature hydrogen absorption kinetics curve is shown in Figure 4. The maximum hydrogen absorption of Zr_2_Fe alloy after different activation treatment times was 1.707 wt %, 1.752 wt %, 1.848 wt %, and 1.840 wt %. It can be seen from Figure 5 that when activating for 1 h, the hydrogen absorption of the material had a certain incubation period. As the activation time was prolonged, the incubation period was shortened to zero, and the maximum hydrogen absorption gradually increased. When the activation time reached 3 h or more, the maximum hydrogen absorption amount was 1.84%, indicating that prolonging the activation time contributed to the activation of the material. When the activation time reaches 3 h or more, the material can be substantially activated completely.

#### 2.1.3. Optimization of the Initial Activation Hydrogen Pressure

The activation time is 3 h at 400 °C, and the hydrogen absorption kinetics at room temperature under different hydrogen pressures (0.1, 0.3, 0.65 MPa) are shown in Figure 5. It can be found that as the initial hydrogen absorption pressure increased, the hydrogen absorption rate gradually increased, while the hydrogen absorption amount decreased gradually. At 0.1, 0.3, and 0.65 MPa, the maximum hydrogen absorption of the materials was 1.848 wt %, 1.406 wt %, and 1.302 wt %, respectively. It can be inferred that at higher hydrogen pressures, the material may be disproportionated.

**Figure 6:**
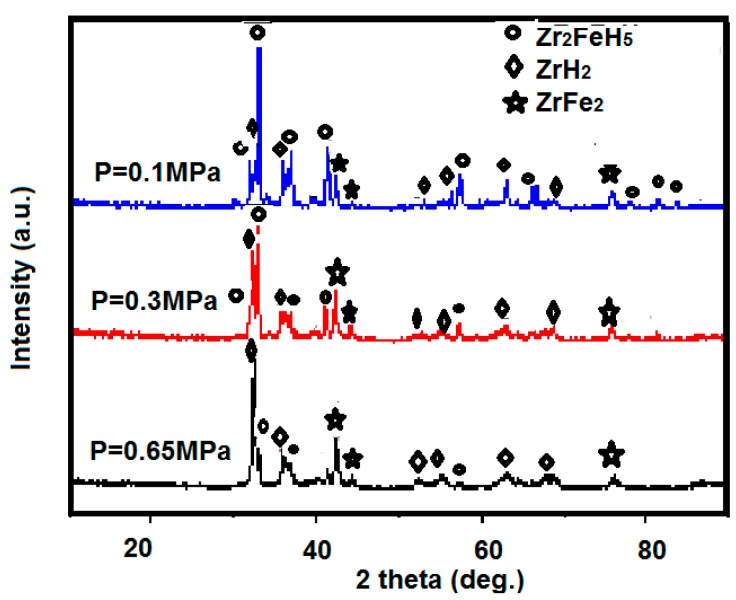
XRD pattern of Zr_2_Fe alloy after hydrogen absorption at different initial hydrogen pressures after activation at 400 °C for 3 h.

By comparing the XRD patterns of the samples after hydrogen saturation at different initial hydrogen pressures as shown in Figure 6, it is found that, under the same activation temperature and time, and under a certain hydrogen pressure, the hydrogen absorption products of Zr_2_Fe alloy form with Zr_2_FeH_5_, ZrH_2_, ZrFe_2_ phase composition. Among them, the hydrogen absorption product under the initial hydrogen pressure of 0.1 and 0.3 MPa is dominated by Zr_2_FeH_5_, and the peak of the main phase of the sample Zr_2_FeH_5_ which absorbs hydrogen under a hydrogen pressure of 0.1 MPa is high, containing only a small amount of other heterophases. The sample after hydrogen absorption at initial hydrogen pressure of 0.65 MPa is mainly composed of disproportionation product phase ZrH_2_ and ZrFe_2_, as shown in the rhombic and empty rhombic shapes in the XRD patterns, respectively. The ZrH_2_ and ZrFe_2_ diffraction peaks are stronger than the sample with an initial hydrogen absorption pressure of 0.3 MPa, which means the disproportionation product is more.

Therefore, the Zr_2_Fe alloy is prone to disproportionation under a certain hydrogen pressure, and the hydrogenation product Zr_2_FeH_5_ undergoes a disproportionation reaction, while hydrogen absorption results in the material not reaching the saturated hydrogen absorption amount.

### 2.2. Disproportionation Properties of Zr_2_Fe Alloy

The above studies show that the initial hydrogen pressure has a significant effect on the hydrogen absorption disproportionation of the Zr_2_Fe alloy. When the hydrogen absorption pressure is slightly higher, the disproportionation product phase is formed. In order to study the effect of temperature on the hydrogenation disproportionation of Zr_2_Fe alloy, the experimental design of this stage is as follows: Firstly, it is activated at 400 °C for 3 h, then cooled to room temperature. Hydrogen is absorbed to saturation at 0.1 MPa hydrogen pressure, and then heated to the set temperature (300–600 °C) to study the disproportionation at different temperatures.

**Figure 7:**
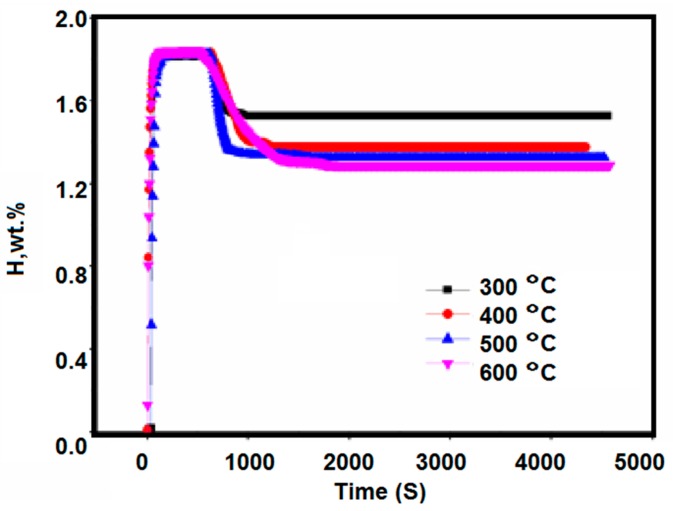
Disproportionation reaction curves of Zr_2_FeH_5_ during 1 h hydrogen release at 300–600 °C.

The experimental results are shown in Figure 7. The hydrogen absorption of the Zr_2_Fe alloy sample at room temperature initially heated up from 300 to 600 °C. The absorbed hydrogen was reduced from the initial saturated state, from around 1.84 wt % to 1.525 wt %, 1.374 wt %, 1.325 wt %, and 1.285 wt %. At the same time, compared with the hydrogen absorption disproportionation curve at 300 °C, it was found that the hydrogen absorption of the final equilibrium of the sample at 400–600 °C was significantly reduced, which is related to the disproportionation reaction of Zr_2_FeH_5_, in addition to the effect of temperature on the saturated hydrogen absorption of Zr_2_Fe alloy. Therefore, it can be inferred that the temperature at which the disproportionation reaction of the Zr_2_Fe alloy starts to occur is about 400 °C. At 300 °C, the disproportionation reaction does not substantially occur.

**Figure 8:**
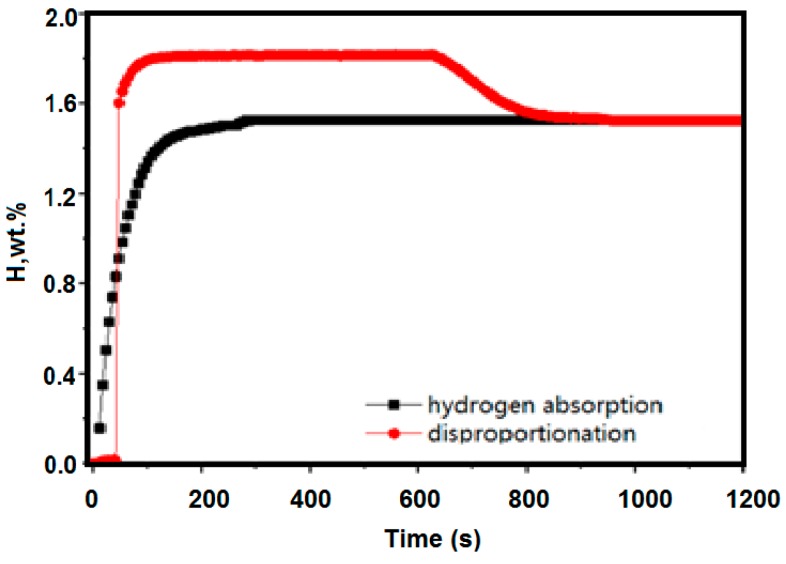
Kinetic curves of hydrogen absorption at 300 °C under hydrogen pressure of 0.1 MPa after activation of the alloy at 400 °C (black line); the disproportionation performance test is 300 °C (red line).

Figure 8 shows a kinetic curve of hydrogen absorption at 300 °C under hydrogen pressure of 0.1 MPa after activation of the alloy at 400 °C. The hydrogen absorption of the material at this temperature is 1.528 wt %, and the disproportionation performance test is 300 °C. Under these conditions, the final stable hydrogen absorption amount is similar, and it can be seen that at 300 °C, no disproportionation reaction occurred substantially.

**Figure 9:**
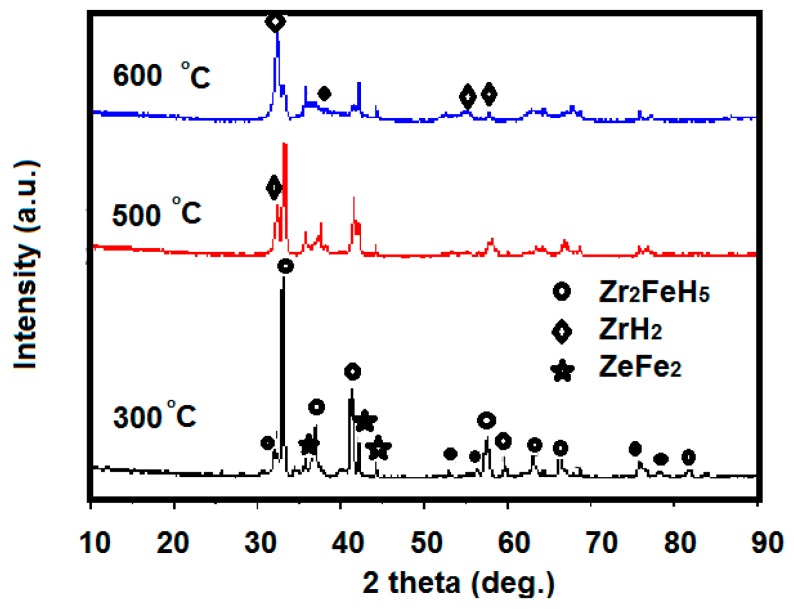
XRD pattern of samples after disproportionation test at different temperatures, after hydrogen absorption at room temperature.

XRD scanning of the sample at 300 °C disproportionation after hydrogen absorption at room temperature is shown in Figure 9. The main phase of Zr_2_Fe alloy after the 300 °C disproportionation test is Zr_2_FeH_5_, but this still contains trace amounts of disproportionation products, ZrFe_2_ and ZrH_2_. The Zr_2_FeH_5_ phase can be maintained substantially at 300 °C, and most of the Zr_2_FeH_5_ does not undergo disproportionation. Comparative analysis of the sample structure after a disproportionation test at 500 °C and 600 °C reveals that as the temperature increases, the disproportionation product increases. The main phase of the sample product at 600 °C changed to the ZrH_2_ phase, meaning that Zr_2_FeH_5_ is completely disproportionate.

## 3. Materials and Methods

**Figure 10:**
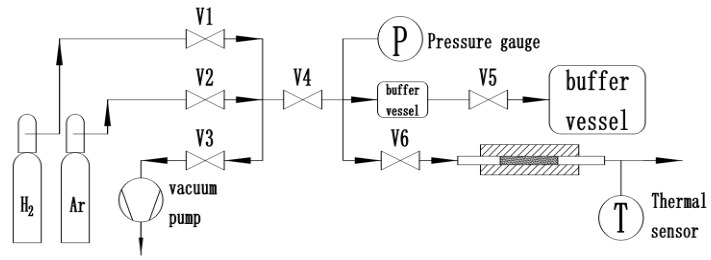
Hydrogen absorption and desorption performance measurement system based on Sieverts’ method.

The Zr_2_Fe alloy was prepared by medium frequency induction melting in the form of granule, with particle size of 100–200 mesh. To test hydrogen absorption and other related properties of the alloy, 2 g samples were loaded in a homemade Sievert-type apparatus, where isothermal kinetic testing was completed on a homemade hydrogen storage material testing system, as shown in Figure 10. The dehydrogenating kinetics measurement was taken by volumetric method. All vials and valves were made of 316 L stainless steel. The furnace temperature was controlled at the accuracy of 0.5 °C. The H_2_ and Ar used in measurement had a high purity of 99.99%. All the de-/hydrogenation measurements were taken in a constant environment, which was achieved by balancing the sample vial and buffer tank volume ratio. In order to avoid disproportionation and to optimize the activation process, we systematically investigated the influence of different activation conditions, especially temperature, time, and hydrogen pressure, on Zr_2_Fe hydrogenation/dehydrogenation and the cooperating effect between hydrogen pressure and temperature on Zr_2_Fe alloy disproportionation.

The crystal structure of the Zr_2_Fe at different stages, especially hydrogen absorption and disproportionation processes, was characterized by a Smart Lab x-ray power diffraction (XRD, Rigaku, Japan) with Cu Kα radiation, 45 kV, and 200 mA. Furthermore, 200 mesh powder XRD patterns were recorded in steps of 0.02° (2theta) from 10 to 90° with a constant scanning rate of 5° min^−1^.

## 4. Conclusions

Zr_2_Fe alloy is promised to be used in the tritium safety systems in future fusion reactors. In this paper, the activation process and hydrogen-induced disproportionation process of Zr_2_Fe alloy were studied. The optimum activation condition of Zr_2_Fe alloy is 400 °C for 3 hours. Zr_2_Fe alloy is better to be used under hydrogen absorption pressure of 0.1 MPa at room temperature. The optimum hydrogenation concentration is 1.84 wt %. The hydrogen disproportionation reaction of Zr_2_Fe alloy under different temperatures and pressure was also studied. The results indicate that no disproportionation reaction occurs under the condition of 300 °C. The hydrogen absorption amount of Zr_2_Fe alloy is 1.528 wt % at 0.1 MPa hydrogen pressure.
